# Recent Advances and Applications of Molecular Docking to G Protein-Coupled Receptors

**DOI:** 10.3390/molecules22020340

**Published:** 2017-02-22

**Authors:** Damian Bartuzi, Agnieszka A. Kaczor, Katarzyna M. Targowska-Duda, Dariusz Matosiuk

**Affiliations:** 1 Department of Synthesis and Chemical Technology of Pharmaceutical Substances with Computer Modelling Lab, Medical University of Lublin, 4A Chodźki Str., PL20093 Lublin, Poland; agnieszka.kaczor@umlub.pl (A.A.K.); dariusz.matosiuk@umlub.pl (D.M.); 2 School of Pharmacy, University of Eastern Finland, Yliopistonranta 1, P.O. Box 1627, FI-70211 Kuopio, Finland; 3 Department of Biopharmacy, Medical University of Lublin, 4A Chodźki Str., PL20093 Lublin, Poland; katarzyna.targowska-duda@umlub.pl

**Keywords:** GPCRs, docking, drug design

## Abstract

The growing number of studies on G protein-coupled receptors (GPCRs) family are a source of noticeable improvement in our understanding of the functioning of these proteins. GPCRs are responsible for a vast part of signaling in vertebrates and, as such, invariably remain in the spotlight of medicinal chemistry. A deeper insight into the underlying mechanisms of interesting phenomena observed in GPCRs, such as biased signaling or allosteric modulation, can be gained with experimental and computational studies. The latter play an important role in this process, since they allow for observations on scales inaccessible for most other methods. One of the key steps in such studies is proper computational reconstruction of actual ligand-receptor or protein-protein interactions, a process called molecular docking. A number of improvements and innovative applications of this method were documented recently. In this review, we focus particularly on innovations in docking to GPCRs.

## 1. Introduction

Recently, one can observe a significant improvement in understanding of structure and function of G protein-coupled receptors (GPCRs). This large family of proteins, which includes as many as 800 members [1,2], is responsible for an essential part of signaling in vertebrates and, as such, invariably remains in the spotlight of medicinal chemistry. GPCRs are responsible for receiving signals transmitted by numerous neurotransmitters, like dopamine, serotonin or acetylcholine, as well as for reception of odors, taste or light. All GPCRs are constructed on the scaffold of seven transmembrane helices, which can be found also in non-GPCR proteins, e.g., bacterial proton pumps [3,4]. It is speculated that the main GPCR families may not be evolutionally related [5] which, together with large number of family members, would suggest that for some reason this scaffold may be optimal for signal transduction. Upon signal reception, usually an extracellular signal is transmitted to the cell interior via receptor conformational changes, and passed on to intracellular messengers, which include G proteins [6,7], arrestins [8] and others [9]. The conformational changes involved in receptor activation involve rearrangements of the transmembrane helical bundle, in particular the 3rd, 5th, 6th and 7th transmembrane helices [10,11,12,13,14,15,16]. Single GPCR can interact with various intracellular partners, depending on the interacting ligand, and selective excitement of a particular signaling pathway is possible with so called functionally selective compounds. Moreover, the signal can be modulated by third-party factors in an allosteric way, i.e., a signal induced by a ligand and received by an intracellular partner can be affected by another influence, acting in a location different than the binding sites of both the ligand and the intracellular partner. A growing number of studies on the mechanisms of activation, functional selectivity or allosteric modulation of GPCRs have resulted in deeper insights in those phenomena, especially since, due to the numerous common structural and sequential patterns present in the entire family or subfamilies, it is suggested that some common mechanisms may be involved [17]. Computational studies play an important role in this process, since they allow for observations at spatial and temporal resolutions inaccessible by other methods. However, all computational approaches are governed by the “garbage in-garbage out” principle, so every step of the molecular modeling process has to be careful and well-validated. One of the key steps is the proper reconstruction of actual ligand-receptor interactions. For instance, a study on allosteric modulation of a receptor requires a correct model of a receptor [18], a modulator, and proper prediction of both the location of the allosteric binding site and modulator interactions at this site. The process of predicting such interactions is called molecular docking. The molecular docking approach has been widely used ever since the early 1980s [19]. It can be applied to identify the ligand binding pocket and to predict the interaction(s) between a small molecule and a protein at the atomic level, as well as to predict interactions between two proteins. Although protein-protein docking is even older than small molecule docking [20] the latter method is much more frequently used [21]. The small molecule-docking process involves: (1) prediction of the ligand position, its conformation as well as orientation within the suggested sites, and (2) ranking these conformations (poses) via a scoring function (corresponding to the ligands’ binding affinity toward the selected target). The second step is at least equally important as the first one—sufficient sampling of the accessible conformational space of protein and the ligand will probably result with at least one pose corresponding well to the actual one, but it is the scoring function that has to sift the correct complex from the large number of all the generated conformations. Therefore, it should be stressed that regardless of the docking algorithm employed, the associated scoring function is an inevitable and crucial component of the process that strongly affects quality of results, for it is the scoring function that has to distinguish hits from the noise of false complexes.

There are various docking strategies, varying in computational cost and exhaustiveness of the conformational search—while both proteins and their ligands are dynamic entities, considering their full flexibility would be very time- and resource-consuming, which makes it unsuitable for many applications. Therefore, a docking strategy is usually chosen to match the aim and available computational resources. A number of available algorithms and strategies were already reviewed [22,23]. However, a number of improvements and innovative applications can be observed recently in this field. For instance, a growing availability of computational power, together with improved algorithms has resulted in applications of molecular dynamics as a docking and/or a scoring tool [24,25]; new editions of molecular docking competitions revealed some new useful tools [26,27,28]; protein-protein docking has become an even more established method for predicting GPCR dimers. There is also visible improvement in the field of peptide-protein docking, which is very difficult due to the considerable ligand flexibility. These improvements are particularly important in computational studies on phenomena as subtle as functional selectivity or allosteric modulation of GPCRs. When small changes in a substituent of the ligand can completely change its signaling bias [8,29], accurate reproduction of protein-ligand interactions becomes crucial. Fortunately, constant development of docking methods, together with improvements and inventions of novel methods of analysis allows for preparing more and more sophisticated in silico studies, making it possible to get deeper insight into such delicate mechanisms [30,31], which is reflected in the growing importance of computational methods in life science.

## 2. An Overview of Docking of Small Molecules to GPCRs

**Figure 1:**
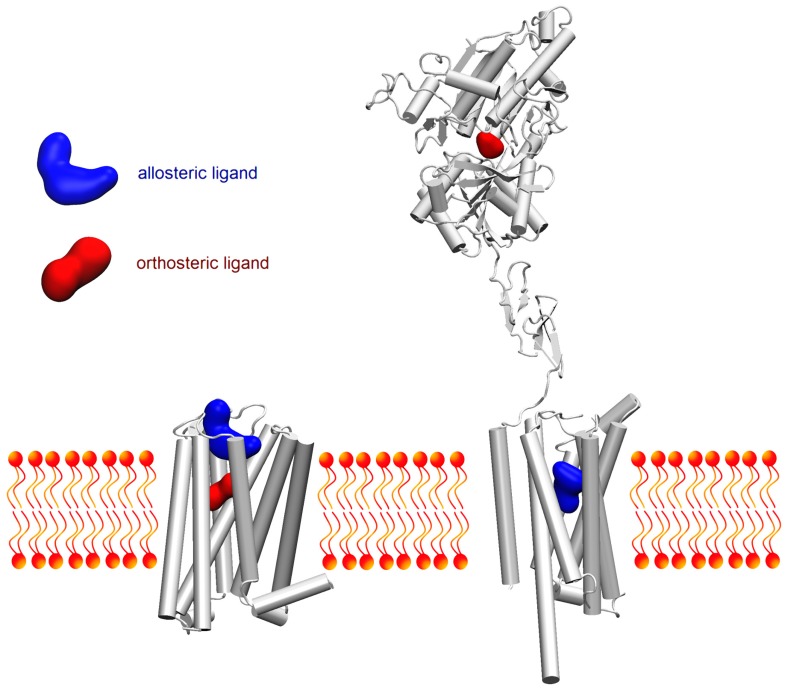
Schematic overview on the most frequent locations of orthosteric and allosteric sites. While in rhodopsin-like receptors orthosteric site tends to be buried inside the transmembrane bundle (left), in most members of other families of GPCRs the orthosteric site is located in large extracellular domains (right). Allosteric sites can be located in various regions of a receptor, but usually small-molecule modulators bind to extracellular vestibule of rhodopsin-like GPCRs (left). In contrast, the most frequently explored allosteric sites in members of other GPCR families are located inside the transmembrane bundle (right), analogically to rhodopsin-like GPCR’s orthosteric sites. M_2_ muscarinic receptor (PDB ID: 4MQT) on the left, mGluR3 and mGluR5 glutamate receptor monomer fragments on the right (extracellular and intracellular, PDB IDs: 2E4W and 5CGC, respectively).

In the case of docking of small molecules to GPCRs, the difficulty of the task greatly depends on the subfamily to which the target belongs. It is typically easier to define a reasonable binding site for aminergic receptors (e.g., 5-HT_1B_ or 5-HT_2B_, as reviewed recently by Michino et al. [32]). The orthosteric binding site in these receptors is formed by a highly conserved Asp 3.32 residue (Ballesteros-Weinstein notation [33]) in the third transmembrane helix (TM3) deep inside the transmembrane bundle. This residue mediates an essential salt bridge with the positively charged nitrogen atom from the ligand. The hydrophobic fragment of the ligand is usually situated between TM3 and TM6 helices (Trp6.48, Phe/Tyr6.51, and Phe6.52). In contrast, other class A [34] and class B, C and F receptors have larger and more open or very lipophilic binding pockets, and this gives more freedom for ligand and makes an accurate prediction extremely challenging (Figure 1). If there is a lack of information in the literature about the binding sites, one should compare the target protein with a family of proteins sharing a similar function or with proteins co-crystallized with other ligands. Moreover, there are available several cavity detection programs and online servers, including POCKET [35], SurfNet [36], PASS [37], fpocket [38], eFindSite [39], Cavitator [40] as previously reviewed by Kaczor et al. [41].

Molecular docking is frequently used in the process of computer aided drug design (CADD). It can be applied in different stages of the drug design process in order to: (1) predict the binding mode of already known ligands; (2) identify novel and potent ligands and (3) as a binding affinity predictive tool. The docking can be conducted by application of a number of docking programs, including AutoDock [42], AutoDock Vina [43], Molecular Operating Environment (MOE) [44], FlexX [45], GOLD [46] and Glide [47]. Different search algorithms are designed to predict the biological activity of studied compounds through the evaluation of the interactions between ligands and potential targets [48].

An increase in the quality of the ligand docking can be gained by consideration of flexibility of the modeled system. There are two major classical docking method categories that allow for a certain degree of receptor flexibility: (1) induced fit docking (IFD) and (2) ensemble docking. IFD is used to search for the new conformational space by the modeling of flexibility of protein (side-chain or limited backbone variations) during the docking simulation. For instance, the IFD methods were introduced by Autodock 4 [49], AutoDock Vina [43] and IFD from Schrödinger, Inc. and successfully applied for serotoninergic 5HT_1A_ and 5HT_7_ receptor modeling [50]. Another interesting method for introducing flexibility to the docking process is using molecular dynamics simulations as a docking tool. This docking strategy is discussed in more detail in a separate section of this review.

The molecular docking results can produce valuable data concerning the location of the binding pocket. However, the docking success rates can be improved by use of available structural (e.g., NMR or X-ray crystallography) data giving insight into favorable ligand conformations [51]. In addition, other experimental data, such as site-directed mutagenesis studies facilitate the prediction of ligand-receptor complexes. The growing number of X-ray structures of GPCRs in complex with different ligands, such as full agonists, partial agonists, biased agonists, and antagonists increases our understanding of the principles of dynamic intramolecular packing in these proteins. However, the different activation states of the receptor induce distinct shapes of the ligand binding pocket as well as contribute to the complexity of the accurate prediction of the possible ligand interactions with GPCRs.

To evaluate the progress in GPCR structure prediction and ligand docking, community-wide GPCR modeling and docking (GPCR Dock) assessments were conducted in 2008 [52], 2010 [53], and 2013 [26]. The main focus of these assessments was the ligand binding pose prediction and its contacts with the surrounding residues of selected targets. The first assessment, GPCR Dock 2008, was focused on the adenosine A_2A_ receptor in complex with the ligand—ZM241385. In this competition twenty-nine participating groups submitted 206 models that show a wide distribution in prediction accuracy of the ligand binding mode (average values of 9.5 Å (s.d. 3.8 Å) for ligand RMSD and 4 (s.d. 7) for the number of correct contacts) [52]. However, very few models score well in both ligand RMSD and the number of correct contacts. The most accurate models were built by homology with the β_2_ adrenergic receptor structure that shares ~35% sequence identity with A_2A_ receptor in the transmembrane domain. The most successful prediction protocols were elaborated using small-molecule docking programs such as GOLD [46], AutoDock [42], Glide [47] and ICM [54].

In the GPCR Dock 2010 assessment, three different classes of receptors were evaluated. In particular, dopamine D_3_ receptor in complex with eticlopride (small ligand pocket); chemokine receptor CXCR4 bound to isoithiourea IT1t (large peptide binding pocket); and CXCR4 bound to the CVX15 peptide (CXCR4/CVX15, the first GPCR complex with a peptide-analogue). The participating groups submitted 275 GPCRs complex models. Correct prediction of the ligand position and the atomic contacts between ligand and the binding pocket was the primary goal of the GPCR Dock 2010 participants. The highest degree of accuracy was achieved in the D_3_/eticlopride model assessment by the group from Pompeu Fabra University. It represented the best prediction in terms of ligand pose (0.96 Å RMSD to the crystal structure) and atomic contacts (58% correct contacts). The ligand is shown in a correct conformation (0.34 Å RMSD after ligand structure superimposition) though translated by ~0.8 Å with respect to its position in the target structure.

In the last GPCR Dock 2013 assessment, four targets, including two human 5-hydroxy-tryptamine (5HT) receptors (5HT_1B_ and 5HT_2B_) in complex with an agonist ergotamine, and a human smoothened homolog receptor (SMO) in complex with LY-2940680 and SANT-1 were selected to evaluate the progress of molecular modeling and ligand docking in context of GPCRs [26]. Many of the submitted models successfully predicted the activation state of 5HT_1B_, but not the biased state of 5HT_2B_. However, the main focus of the assessment for these receptor targets was prediction of the binding pose of the ligand and its contacts with the surrounding residues. For the 5HT_1B_/ergotamine complex, the median ligand RMSD was 5.44 Å, with the best result as low as 1.5 Å. The median contact prediction accuracy was equal to 15.1%, the best being 51.1%. The best RMSD and the best contact prediction were achieved in two models presented by the Stockholm Carlsson group (Stockholm University). In the case of the 5HT_2B_/ergotamine complex the median ligand RMSD was 5.54 Å and the best being 1.05 Å (model presented by the group from Warsaw); the median contact accuracy was equal to 17.8%, and the best being 53.1% (achieved in the model presented by the Stockholm-Carlsson group) [26]. Moreover, the correctness of the best ergoline core (its interactions closely resemble those observed in other biogenic amine receptors) predictions corresponds to 13.01% and 19.55% of the experimental distribution in the 5HT_1B_ and 5HT_2B_ assessments, respectively [26]. Finally, for the SMO/LY-2940680 complex the top scoring model (presented by the group from Vanderbilt University) weakly resembles the experimental structure (4.42 Å of the ligand deviation and no strong correct contacts (contact accuracy of 2%)). However, marginally better contact prediction was achieved for SMO/SANT-1 complex (the top-ranking models were presented by the group from Georgia Institute of Technology (contact accuracy of 7%), UMich-Zhang group from University of Michigan (11% of contacts captured), and the group from Seoul National University (contact accuracy of 12%) [26].

### Recently Introduced Docking Algorithms

Since there have been no GPCR Dock competitions since 2013, a number of novel methods were not ranked with respect to GPCRs. However, a number of other competitions were performed, giving an overview on general quality of a number of these methods. For instance, the CSAR 2014 Exercise included docking to a number of X-ray structures [27], while the D3R Grand Challenge 2015 ranked docking and scoring protocols on the basis of their performance on two datasets, consisting of HSP90 and MAP4K4 proteins [28]. Still, not all the novel algorithms have been ranked in any competition. However, some of them are interesting and promising, and therefore, we decided to include some of the most recent reports, even if they were not yet tested on GPCRs.

One of such recently released algorithms is Yada [55]. It is a freely available tool reported in September 2016. It is a genetic algorithm designed to perform well in blind docking. For this purpose, Yada utilizes biological information, e.g., it considers presence of conserved residues during a binding site detection. Moreover, water molecules are also taken into consideration.

The first edition of D3R Grand Challenge was an opportunity to prove the usefulness of DockBench [56]. It is a tool designed to automatically compare a number of docking procedures. Just as the previous tool, DockBench is available free of charge.

An interesting application of docking to investigate enzymes that bind their substrates covalently is presented by DOCKovalent [57]. Although invented for enzymes, the method could be useful also in the field of GPCRs, e.g., for in silico elaboration of experimental results of substituted cysteine accessibility studies [58].

Some new algorithms and applications tackling the problem of peptide ligands and their huge flexibility were also reported. These reports affect the GPCR field directly or indirectly, since there is a number of these receptors binding peptides as native ligands. The most characteristic feature of one of such new algorithms, AnchorDock, is incorporation of ligand recognition mechanisms into the docking process [59]. It performs folding of the peptide, and simultaneously search for suitable binding sites. This strategy is less time-consuming than more traditional methods. Another new fully blind docking approach, called MDockPeP, first generates peptide structures on the basis of the template fragments and then performs modeling of the protein-peptide complex, which resembles classical docking [60]. In turn, knowledge-based strategy was implemented in GalaxyPepDock [61]. The program gathers information from databases of known protein-peptide interactions, and it is freely available as a web server. Certainly, these new algorithms improve the quality of peptide docking results and/or require less computational resources than classical ones. However, a recent benchmark prepared by Hauser and Windshügel suggests that some of the more established algorithms like Surflex [62] or AutoDock Vina [43] can also perform well in docking of peptides longer than five aminoacids [63].

## 3. Modeling of Water Molecules within the Binding Site

Water molecules can be often found in the proximity of ligands in crystal structures of proteins. One well known example is the X-ray structure of the μ opioid receptor (MOR) in its inactive state (PDB ID: 4DKL), where an interaction of the phenolic group with His6.52, which is important for opioid ligand binding, is mediated by two water molecules [64]. Efficient and reliable calculations of the effects of water on ligand binding are still a challenge. Solvation effects are of key importance for lead optimization where a 100-fold difference in binding affinity is significant but corresponds to only ~3 kcal/mol in binding free energy [65]. Protein mobility is most probably increased by the presence of moving and displaceable water molecules inside and outside the protein and, in particular, within the binding site [66]. Water molecules are in fact critical in binding and recognition process. They are responsible for discrete hydrogen bonded water networks, hydrophobic effects and they are also involved in the stabilization of protein-ligand complexes [66]. As it was mentioned, modelling of water-mediated effects is difficult and a number of diverse approaches, starting from continuum solvent models up to methods considering waters as explicit components or at a quantum level have been developed [66,67].

Multiple approaches to compute the significance of water in structure-based drug design and to predict its energetic contributions have been developed. It was demonstrated that including crystal water in virtual screening improves the screening results [68]. The software used for modelling water molecules include GRID [69], HINT (Hydropathic Interactions) score and geometric Rank descriptor [70], Superstar [71], Just Add Water Molecules (JAWM) [72], WaterMap [73], Water PMF [74] and Water FLAP [75] (Table 1). In addition, some molecular docking software is able to consider the presence of water molecules at least to some extent (GOLD: estimation of the free-energy change associated with transferring a water molecule from the bulk solvent to its binding site in a protein-ligand complex, DOCK: flexible-receptor docking method considering displaced water and retained water states with variable water position, FlexX: particle concept, AutoDock: hydration force field accounting for the entropic and enthalpic contributions of discrete waters to ligand binding, Glide: statistics about the number of hydrogen bonds formed by polar and apolar groups [66]), see Table 1. Recently, Kim and Cho developed a new docking protocol which incorporates quantum mechanical/molecular mechanical (QM/MM) calculations along with an implicit solvent model [76]. An interesting approach has been also reported by Lenselink et al. [77] who investigated the importance of explicit water molecules in virtual screening, taking as an example the adenosine A_2A_ receptor, where water molecules have previously been shown to be important for achieving high enrichment rates with docking, and where the positions of some binding site waters are known from a high-resolution crystal structure. They assessed the influence of the water molecules (regarding both their presence and orientations) on virtual screening (VS) enrichment applying carefully curated set of 299 high affinity A_2A_ antagonists and 17,337 decoys. They demonstrated that including certain crystal waters greatly improves VS enrichment and that optimization of water hydrogen positions is needed in order to achieve the best results. Moreover, they also proved that water molecules derived from MD simulations without any prior knowledge of crystallographic water molecules are capable of improving enrichments to a similar degree as with the crystallographic water molecules.

In particular, WaterMap (Schrödinger) is an efficient approach for calculating the cost of desolvation for structural waters [78]. WaterMap performs an all atom explicit solvent molecular dynamics simulation which is then followed by a statistical thermodynamic analysis of water clusters [79]. WaterMap waters which are predicted to have a considerable positive free energy relative to being in bulk solvent are termed ‘unhappy’ [79]. These water molecules should be displaced by ligands thus the relative position of the ‘unhappy’ water clusters computed by WaterMap reveal hotspots for small molecule ligand binding [79]. The role of water molecules, e.g., during GPCR activation process [80] can be also investigated using molecular dynamics. It should be stressed that application of WaterMap leads to results which are consistent with the results of molecular dynamics simulations. WaterMap was applied to find that hydration site thermodynamics explain SARs for triazolylpurines analogues binding to the adenosine A_2A_ receptor [81]. Recently, Schrödinger has introduced a new scoring function that includes water displacement [82]. WScore is a new approach for protein-ligand docking and scoring, which includes a flexible description of explicit water molecules. The locations and thermodynamics of the water molecules are based on WaterMap MD simulations. The water structure is used to supply description of ligand and protein desolvation on the atomic level.

## 4. High-Throughput Docking as a Method of Virtual Screening

Virtual screening is nowadays a method of choice to search large databases of compounds and to select compounds for in vitro testing. Virtual screening approaches can be divided into ligand-based and structure-based. When the 3D structure of a target is known from experimental or computational studies, high-throughput docking is a method of choice. The alternative method is pharmacophore-based virtual screening.

For GPCRs reported crystal structures are now applied in virtual screening to create homology models for close homologs. Indeed, results from several studies have demonstrated that homology models of GPCRs are suitable for structure-based virtual screening [83]. Moreover, high-throughput docking results against GPCR structures are considerably better when compared to results for water-soluble proteins [84]. In virtual screening, the docking hit rates for water-soluble protein are approximately 5%–10% of the molecules tested, resulting in hits with micromolar to mid-micromolar affinity [84]. These hit rates for soluble proteins, in spite of being better than average HTS hit rates are still lower than those of docking ligands to GPCR. This high success rate can be attributed to the fact that even unbiased libraries, such as ZINC, in fact possess a high number of compounds that resemble GPCR ligands. In addition, the well-buried GPCR orthosteric sites can almost entirely sequester or complement a small organic compound, to enable them to be recognized with high ligand efficiency [84]. A number of studies investigated the efficiency of GPCR high-throughput virtual screening exercises [85,86,87] showing that both homology models and crystal structures can be effectively used. An overview of recent successful structure-based ligand discovery and design studies demonstrates that receptor models, in spite of structural inaccuracies, can be effectively applied to search for GPCR ligands [88]. Some successful examples of virtual screening involve the study of a set of GPCRs by Sanders et al. [89], dopamine D_1_ and D_2_ receptors by Kołaczkowski et al. [90], dopamine D_2_ receptor by Kaczor et al. [91], serotonin 5-HT_2A_ receptor by Gandhimathi and Sowdhamini [92], dopamine D_3_ receptor by Carlsson et al. [93], neurotensin receptor 1 by Zhang et al. [94], nociception receptor by Daga et al. [95] and many others.

In order to prepare for virtual screening, it is often necessary to tailor a docking protocol in such a way that it results in a correct ranking list of best compounds. Weiss et al. [96] created GPCR-Bench, a publically available docking benchmarking set in the spirit of the DUD and DUD-E reference data sets for validation studies, including 25 non-redundant high-resolution GPCR co-structures with an accompanying set of different ligands and computational decoy molecules for each target. Benchmarking sets are commonly applied to compare docking protocols; however, it should be stressed that it is necessary to evaluate docking methods not by “retrospective” hit rates but by the actual probability that they will result in new promising hits [96]. This is why docking protocols should not only rank active compounds highly but also create reasonable poses that a scientist will choose for purchase and screening [96].

A widely-used method for identification of hits and leads for targets where structural information is available is to screen fragments. Fragment-based virtual screening is based on finding small chemical fragments, which may bind only weakly to the subpockets of a given target, and then growing them or combining them to produce a lead with a higher affinity. Fragment-based drug discovery (FBDD) has caused a revolution in the process of drug discovery and design, with many FBDD leads being developed into clinical trials or approved in the past few years [97]. Compared with traditional high-throughput screening, it displays obvious advantages such as efficiently covering chemical space or achieving higher hit rates. For example, the performance of an agonist-bound A_2A_ adenosine receptor structure was evaluated in retrieval of known agonists and then employed to screen for new fragments optimally fitting the corresponding subpocket [98]. In another interesting work, Sirci et al. used virtual fragment screening using molecular fingerprints derived from a unique set of fragment affinity data for the histamine H_3_ receptor (H_3_R), a pharmaceutically relevant GPCR [99]. Ranganathan et al. used fragment-based virtual screening to search for subtype-selective adenosine receptor ligands from homology models [100]. De Graaf et al. [101] developed and validated a customized structure-based virtual fragment screening protocol against the recently determined human histamine H_1_ receptor crystal structure. Their method is based on a combination of molecular docking and a protein-ligand interaction fingerprint scoring method. Moreover, Chen et al. [102] studied the potential to complement NMR-based biophysical screening of chemical libraries with molecular docking in fragment-based lead discovery against the adenosine A_2A_ receptor, a drug target for inflammation and Parkinson’s disease. Finally, Vass et al. [103] used virtual fragment screening to identify ligands of dopamine D_3_ and histamine H_4_ receptors.

Nowadays, virtual screening can be also used to search for ligands tailored for distinct conformational states of GPCRs [104]. In this context Tarcsay et al. [105] suggested that MD simulation is able to capture protein conformations which are less biased toward the binding points of the chemotype that has been crystalized to obtain the X-ray structure. They demonstrated that selected frames from the MD trajectory can perform better than X-ray structures and homology models in terms of enrichment factor and AUC values. Moreover, Kohlhoff et al. [106] used MSM on MD data obtained by cloud-computing approaches which can be used to gain insights into the conformational space and activation mechanisms of GPCRs. The resulting ensemble of conformations can be a starting point for structure-based discovery methods to design compounds that interact more closely with diverse receptor states, for overall increased efficacy and specificity [106]. Bhattacharya and Vaidehi [107] used coarse grain computational methods to understand the modulation of the potential energy landscape of the β_2_ adrenergic receptor by two full agonists, two partial agonists, and an inverse agonist, starting from the receptor crystal structure in complex with an inverse agonist, carazolol. Virtual screening with a salbutamol-stabilized conformation exhibited enrichment of non-catechol agonists over a norepinephrine-stabilized conformation which was produced by a different activation pathway [104]. Gandhimathi and Sowdhamini performed virtual screening and docking studies with both active and inactive state models of serotonin 5-HT_2A_ receptor obtaining agonist-like and antagonist-like molecules [92]. Computer-aided drug design aimed at discovering biased agonists has still not been extensively exploited [104]. However, one approach has been shown to be a prospective method in the blind prediction contest GPCR Dock 2013 [26]. A major challenge of the mentioned Dock 2013 was to find the binding mode of ergotamine, a biased agonist of serotonin 5HT_2B_ receptor which causes full β-arrestin-mediated response but only partial G protein activation. The methodology was based on ligand-guided homology modelling, which links extensive molecular docking screening with information from a number of crystal structures and experimentally derived restraints [34]. Rodriguez et al. [108] retrospectively analyzed thousands of structures that were generated during this assessment to evaluate their modeling strategies. Major improvements were identified as modeling of extracellular loop two in combination with the use of molecular docking to optimize the binding site for ligand recognition. Their results indicated that modeling of GPCR-ligand complexes is now so accurate that structure-based drug design could be applied to a large number of pharmaceutically relevant targets. In the context of agonist identification using structure-based virtual screening, Weiss et al. [109] carried out large library virtual screen against an activated β_2_-adrenergic receptor structure which resulted in potent agonists to the exclusion of inverse-agonists, being the first complement to the previous virtual screening campaigns against inverse-agonist-bound GPCR structures, which identified inverse-agonists only.

Another challenge is to search for selective ligands using structure-based virtual screening. Schmidt et al. [110] identified modulators of chemokine receptors 3 and 4 with tailored selectivity using multi-target docking. Rodriguez et al. [111] reported structure-based discovery of selective serotonin 5-HT_1B_ receptor ligands.

Virtual screening can be also used to identify allosteric modulators of GPCRs as it was done for muscarinic receptors [112] and metabotropic glutamate receptors [113]. Other successful examples of structure-based discovery of GPCR allosteric ligand include identification of GPR68 modulators by Huang et al. [114], and muscarinic M_2_ receptor modulators by Miao et al. [112]. Extension of virtual screening methodology to work on multi-target drug design and polypharmacology is an increasingly significant aspect in drug design [115]. New interaction fingerprint techniques enable structural chemogenomics and polypharmacology predictions by complementing the increasing amount of GPCR structural data [116].

In summary, virtual screening is a technique capable of reducing both time and cost through reducing the number of compounds which have to be experimentally tested in order to identify hits which bind to biological targets of therapeutic relevance [86]. It can be expected that a growing amount of structural information on GPCRs will result in next success stories on high-throughput docking against these receptors.

## 5. Docking by Molecular Dynamics Simulations and Their Modifications

Molecular dynamics simulations are an interesting tool that allows for investigation of events occurring in spatial and temporal scale not available in experimental methods. As such, it can also be used to simulate ligand binding as well as dimerization/oligomerization processes. Therefore, MD could be used as a flexible docking method, characterized by a specific conformational sampling philosophy. Instead of imitating elasticity via creation of conformational ensembles of both ligand and a protein, with subsequent selection of the most favorable poses according to a scoring function, the conformations are created in subsequent steps of a simulation, according to the calculated forces and velocities. This could be considered as a docking and scoring procedures occurring simultaneously, with a continuous scoring based on the force field calculations. On one hand, in this situation conformations are not generated randomly, but they are a response to the calculated mutual ligand-protein interactions, which is probably a better representation of actual conformational selection and/or induced fit events. On the other hand, the created conformational ensemble is not exhaustive, and a ligand can be trapped in a suboptimal well of potential. These problems can be overcome in many different ways, depending on the nature of the investigated problem. There are various examples of the approach in recent literature, using different modifications of a method. In general, due to computational cost of the approach, using MD as a docking tool is reasonable when small number of ligands is considered, and the exact location of the binding pocket is unknown, or the data on the pathway of the ligand to the pocket are essential. MD-based approaches are also the methods of choice in unraveling hidden binding pockets, which are buried in static X-ray structures or molecular models, yet can be exposed during a simulation of a protein-ligand interactions [117,118,119,120,121]. Also, MD or its modifications can also be useful in validation of docking poses obtained with other methods.

The most obvious, but also the most demanding application of MD to docking would be simulation of a receptor with a ligand placed randomly in a simulation box. This would require a considerable amount of simulation time for a ligand to be caught by a receptor, and to find its position in the binding pocket. This strategy was proven to be reliable by long-timescale simulations successfully reproducing ligand positions known from the X-ray structures. Such simulations were performed for GPCRs, e.g., Dror et al. were able to reconstruct the entire process of spontaneous alprenolol binding to the β_2_ adrenergic receptor, with most of the binding poses showing RMSD as low as 0.8 Å from the crystallographic pose [24]. Also proteins other than GPCRs, like enzymes [122,123] were subjects for such reconstructions. Such successful studies on orthosteric ligand binding encouraged researchers to attempts of allosteric binding site predictions. In October 2013, a work of Dror et al. on prediction of allosteric binding site in M_2_ muscarinic acetylcholine receptor was published electronically [124]. The paper pointed out some key residues responsible for the modulator binding, like Trp 7.35, Tyr 2.61 or Tyr 2.64. Interestingly, as quickly as a few weeks later a paper of Kruse et al. appeared, where they announced a release of the 4MQT X-ray structure of the M_2_ receptor with an allosteric modulator [125]. The structure confirmed the predicted localization and the key contacts responsible for binding of allosteric modulators. These particular studies were a valuable proof of MD’s usefulness in binding site prediction, even when only the weak interactions at exposed allosteric sites are in play. This was both an encouraging stimulus for further applications, and an argument supporting validity of hypotheses derived from earlier MD studies [126]. Among such interesting earlier studies there were, for instance, all-atom unbiased simulations of a lipophilic ligand entering a cannabinoid receptor from the membrane side [127]. However, a study of Kruse et al. demonstrated, that some binding events require significant simulation time, and some of them couldn’t be reproduced even using the Anton supercomputer [128,129].

After the encouraging success of Dror et al., MD was much more frequently used as a high-quality docking method, particularly useful in finding allosteric binding sites. However, instead of all-atom unbiased MD used in that study, various modifications are usually used. Such methods were known and used for docking for some time, e.g., in a study of Provasi et al. on binding of naloxone to the δ opioid receptor [130], or in a study of Sabbadin and Moro, who investigated binding of various ligands to the adenosine A_2A_ receptor [131]. However, these studies utilized mostly the ligand-receptor configurations of widely accepted binding modes, and focused mostly on binding mechanisms and ligand entering pathways. At present, both all-atom, unbiased MD, as well as modified MD, like accelerated molecular dynamics (aMD) [132] or coarse-grained MD (cgMD) become more and more frequently used as a full, sophisticated docking tool. For instance, all-atom unbiased simulations of the opioid receptor with high concentration of its G protein-biased ligand, TRV-130, were used in search of its binding mode [133]. Recent aMD applications include a study of Kappel et al., where authors were able to simulate a process of ligand binding to acetylcholine M_3_ receptor [134].

An interesting application of aMD in the virtual screening workflow was recently reported by Miao et al. [112]. In their study, simulations were used for building an ensemble of receptor conformations only, and further steps were performed with other docking tools. This strategy is an interesting improvement of known flexible docking algorithms, and resulted in successful design of active substances.

Coarse-grained MD can be used to investigate GPCR dimerization or oligomerization. This application was reviewed recently [21]. Interestingly, some of such recent studies balance on the edge of protein-protein and protein-small molecule docking. For instance, cgMD was recently used by Prasanna et al. in a study on the influence of cholesterol membrane concentration on the GPCR dimer formation [135]. Except of indirect effects, some specific cholesterol occupancy regions at serotonin 1A receptors were identified. The authors were able to identify an increased occupancy at the 6th transmembrane helix, which is known to be involved in GPCR activation. Another cgMD approach to the problem of cholesterol binding and receptor dimerization was undertaken by Pluhackova et al., using CXCR4 receptor immersed in membranes of various cholesterol concentrations [136]. In this case, the highest occupancies were observed at the TM1-TM7 interface. On the other hand, Khelashvili et al. addressed the problem with all-atom MD of rhodopsin. They observed a number of highly occupied sites, including extracellular ends of TM2-TM3, intracellular ends of TM1-TM2-TM4, and a site at TM7.

MD is sometimes used in combination with other docking tools. In such cases, MD is responsible for validation and/or refinement of docking poses. Using a representative set of initial docking positions, subsequently subjected to MD is a convenient way to reduce the required computational resources in comparison to simulations of entirely spontaneous binding, while maintaining most of the predictive value. Such strategy was employed in studies of Bartuzi et al. on the allosteric modulation of the µ opioid receptor [31,137]. A number of putative allosteric sites was indicated by simple flexible docking, and all these pockets were further investigated by all-atom, unbiased MD. Only little fraction of docking poses turned out to be stable, and in some simulations modulators migrated convergently to the most stable sites. The suggested location of allosteric binding sites for positive and negative allosteric modulation was also validated with MD, and number of phenomena consistent with experimental data were observed. Very similar allosteric site in a closely related δ opioid receptor was suggested in a metadynamics-based study of Shang et al. [138], which suggests a presence of a common allosteric site in opioid receptors. A similar strategy, more focused on classical docking, but supported with MD was also used in a study of Kastner and Izaguirre, who investigated binding modes of a ligand in the octopamine receptor [139]. Thanks to the approach, an alternate binding mode of the agonist was identified. MD was also used as a support in a study of Milanos et al. [140]. Although all-atom MD failed to unravel any stable binding sites for the investigated ligands, the subsequent metadynamics approach resulted in finding some favorable contacts.

There are also some advances in application of MD to scoring of protein-ligand complexes. An interesting work of Vuong et al. presents possibility of utilizing steered MD (sMD) for evaluation of ligand binding affinity [25]. The study underlines the importance of the ligand pulling direction in accuracy of affinity prediction. Another recent study, reported by Okimoto et al. puts an emphasis on pulling velocity [141]. Both studies conclude that sMD can be efficiently used in estimation of binding affinity, which is clearly an advance when compared to previous studies, where only discrimination of binders from non-binders was possible [142]. Right next to steered MD, the free energy calculations emerge as potential efficient tool for predicting binding affinities. In a recent study, Lenselink et al. presented a consistent protocol involving free-energy perturbation calculations, allowing for satisfactory prediction of properties of novel ligands [143]. While Vuong et al. were able to rank ligand affinities on the basis of pulling forces, Lenselink et al. proposed ranking on the basis of ligand binding free energies. This allowed for successful prediction of binding affinities for 39 of 45 compounds investigated on four target GPCRs.

All the described studies prove the usefulness of MD and its modifications as molecular docking tools. The main drawbacks of such applications are the computational cost and complexity of the proper simulation box preparation. These issues make applications of MD to ligand screening difficult. However, an increasing availability of high-end servers, as well as well performance of even relatively small GPU stations [139] and rational application of MD modifications, such as aMD can make MD-based methods an interesting alternative to classical docking algorithms, especially to the more sophisticated and time-consuming IFD methods.

## 6. Protein-Protein Docking as a Method to Model GPCR Dimers

According to the classical approach, the functioning of GPCRs is described by the ternary complex model as an interplay of three elements: receptor, agonist and G protein. In this model, the activation of the receptor, resulting from interactions with the agonist leads in turn to receptor-G protein interactions in intracellular region and to initiation of specific signaling cascades. However, more and more experimental evidence indicates that signal transduction through GPCRs may be much more complex than the one contemplated in the ternary complex model. Although it was first thought that GPCRs function as monomeric entities, more and more experimental and computational evidence confirms that these receptors form functional homomers and heteromers [144]. The possibility of GPCR dimerization and oligomerization is one of the factors which complicates the process of signal transduction through these receptors [145]. Many cases of homo- and heterodimerization of GPCRs have been described and some of them may lead to a new pharmacological entity with different properties than the separate monomers [146]. This feature, accompanied by the reports about selective distribution of dimers in particular tissues may lead to more selective drugs which act only in the desired places in the organism.

Computational techniques [41,147] are complementary to biochemical and biophysical techniques [148] (e.g., FRET and BRET [149]) for studying GPCR oligomerization. They can be classified to structure-based approaches [21] and sequence-based methods. Structure-based methods include protein-protein docking, molecular dynamics simulations including all-atom molecular dynamics and coarse grained molecular dynamics [150], Normal Mode Analysis (NMA) and de novo design for prediction of the dimerization interface. Sequence-based methods can be divided into two groups: the methods which assume that the dimerization interface is evolutionary conserved (Evolutionary Trace (ET) method, Correlated Mutation Analysis (CMA), or Subtractive Correlated Mutation (SCM)) and the methods that assume that the dimerization interface changes during evolution process (Differential Evolutionary Trace (DET), Spatial Cluster Detection (SCD) and Hidden-Site Class Model of Evolution) [147]. In spite of the fact that sequence-based methods supply valuable information about interface forming residues, it should be stressed that in contrast to structure-based methods they do not result in 3D dimer structures. In case of protein-protein docking, the advantages are connected with the speed of the method and relatively low consumption of computer resources. The advantage of molecular dynamics simulations is the possibility to study dynamic aspects of dimerization, e.g., conformational changes connected with this process. Moreover, molecular dynamics simulations allow to consider the presence of cell membrane. However, it is necessary to remember about the limitations of this technique resulting from time scale and limitations due to time and resource consumption. It should be stressed that protein-protein docking is not as commonly used for modeling GPCR dimers as e.g., molecular dynamics simulations.

In the case of protein-protein docking as applied to GPCR dimers, rigid-body docking is one of the main disadvantages in view of the highly flexible GPCR regions (intracellular and extracellular loops) which may take part, at least to certain extent in GPCR dimer formation [151,152]. Moreover, conformational changes in transmembrane and loop regions upon dimer formation cannot be satisfactorily considered by the rigid approach. Thus, it is very important to take into consideration protein flexibility (at least partially) [21]. Although it was not possible with early protein-protein docking tools, now at least side chains flexibility can be taken into account. Other problem that arises from application of protein-protein docking approach is not considering desolvation terms.

Another significant limitation is that commonly applied docking algorithms and methods to quaternary structure predictions were elaborated mainly for water-soluble proteins, i.e., assume a water sphere around the protein. However, GPCRs are present in a lipid environment of a cell membrane. As a result, widely applied scoring functions are not fully tailored for modeling GPCR dimers. Thus, it was necessary to investigate the applicability of protein-protein docking software for transmembrane proteins, in particular for GPCR dimers. In this context, Kaczor et al. [153] studied eight protein-protein docking tools, i.e., ZDOCK, ClusPro v.1.0, HEX, GRAMM-X v.1.2.0, PatchDock (version beta 1.3), SymmDock (version beta 1.0) and HADDOCK. They selected multimeric transmembrane proteins with known crystal structure deposited in PDB database. In all the experiments they obtained 10 models that were characterized with B_RMSD (the lowest RMSD in comparison to the crystal structure) and A_RMSD (the average RMSD in comparison to the crystal structure). In addition, they determined CAPRI parameters and structural parameters, such as complex surface area, interface area, and polar and hydrophobic contributions to complex surface area and interface area. The analysis of results regarding B_RMSD indicated that the best docking results were obtained with GRAMM-X (median 0.27 Å) and ZDOCK (median 4.20 Å). The values of median of A_RMSD were similar for all the studied tools (11–13 Å). It can be concluded that best protein-protein docking tools result in a few correct models which need to be separated from a great number of incorrect models. Thus, it is not problematic to obtain a correct model but it is a challenge to select scoring functions for the obtained population of models which place the correct models on the top of the ranking list. The main advantage of GRAMM-X over other tools may be connected with the presence of evolutionary conservation score (for interface) in a scoring function in this tool only. Kaczor et al. [153] also analyzed structural features of transmembrane proteins which facilitate or hamper the application of protein-protein docking approach for construction of their complexes. As it can be expected, it is easier to model transmembrane protein complexes with large interface which is rich in cavities that fit to each other. This explains the lack of success in application of protein-protein docking technique to GPCR dimers. Indeed, regarding modeling GPCR dimers, they obtained either incorrect models according to CAPRI criteria (PDB IDs: 4DJH and 3CAP) or only acceptable models (PDB ID: 3OE9). It is also well-known that if the complex formation is accompanied by a significant conformational change, protein-protein docking approach is not able to consider this change in conformation. In this context Duarte et al. [154] found that the putative dimer interfaces proposed for class A GPCRs do not show the usual patterns of stable biological interfaces, neither in terms of evolution nor of packing, thus they likely correspond to crystal interfaces. However, the possibility that they constitute transient or weak interfaces cannot be ruled out. In contrast they observed a clear signature of biological interface for the proposed dimer of the class F human Smoothened receptor. The results obtained by Kaczor et al. [153] indicate that protein-protein docking should be used with great care when applied to modeling GPCR dimers. Moreover, the main problem is to select a correct model from a large population of obtained models. It can be facilitated e.g., by taking into consideration the degree of evolutionary conservation of the interface.

In spite of these limitations protein-protein docking has been used several times for modeling GPCR dimers. GRAMM was applied by Soulier et al. [155] to model the serotonin 5-HT_4_ receptor homodimer for designing bivalent ligands. They found helices TM2,4-TM2,4 and TM4,6-TM4,6 as dimer interface. Fanelli et al. [156] used ZDOCK and subsequent membrane filter to model thromboxane A2 receptor isoform α homodimer. The best model represented a complex in which helix TM1 formed the interface. Kim and Jacobson [157] used the semi-empirical oligomer structure of rhodopsin as a template to establish the distance between monomers in the initial A_3_R-A_3_R dimeric models. The largest interface between two monomers was identified for helices TM4,5 which demonstrated also a high degree of shape complementarity. In addition to GPCR homodimers, some heterodimers quaternary structure has been also predicted: e.g., the adenosine A_2A_ receptor and the dopamine D_2_ receptor heterodimer [158] and the μ and δ opioid receptor heterodimer [159]. A protein-protein docking technique with ROSETTA without a membrane topology filter was applied to build the heterodimer of the mGluR2-5HT_2A_ receptors [160]. However, in this case it was not possible to indicate the correct model using scoring results, thus the output was evaluated according to visual inspection based on experimental evidence resulting in the TM4,5-TM4,5 heterodimer model.

In order to enhance the application of protein-protein docking for modeling GPCR dimers, Kaczor et al. [161] elaborated a protein-protein docking-based protocol with external scoring for this purpose and tested it by an attempt to reconstruct GPCR dimers 3D structure for dimers with known crystal structure deposited in PDB database. They selected six known GPCR dimers and generated preliminary populations of models for them by rotating one monomer around the other with increments of 30 degree which resulted in 144 models for each dimer. Next, they used local protein-protein docking with Rosetta to refine models obtaining 10 models for each orientation which resulted in 1440 models for each studied dimer. The most important element of the protocol was external scoring using 11 scoring parameters: (1) energy of electrostatic interactions at the interface; (2) the degree of evolutionary conservation of the interface; (3) fractal dimension as a measure of surface roughness; the interface should be as smooth as possible [162]; (4) free energy of binding; (5) the contribution of hydrogen bonds to free energy of binding as these bonds are particularly strong and important in hydrophobic environment of cell membrane; (6) interface area; (7) polar contribution to interface; (8) potential energy; (9) Rosetta total score; (10) Rosetta interface score; (11) shape complementarity. Based on their results they excluded some interfaces as improbable for GPCR dimerization. As an example, TM2-TM3-TM2-TM3 interface was excluded which can be justified by the fact that TM2 and TM3 are hidden within a helical bundle and are responsible for interactions with orthosteric ligands or e.g., sodium ions and are not exposed as possible dimerization interface. Moreover, TM3-TM4-TM3-TM4 and TM6-TM7-TM6-TM7 as well as their asymmetric combinations are also not very probable. TM1-TM2-TM1-TM2, TM4-TM5-TM4-TM5, TM7-TM1-TM7-TM1 and their asymmetric combinations with different partners were found to be probable dimerization interfaces. One of the most important conclusion from this work was to indicate the scoring parameters which result in a correct ranking list for dimers with different dimerization interfaces. They postulated application of four scoring parameters for future applications: Rosetta interface score, interface area, free energy of binding and hydrogen bond contribution to free energy of binding. Kaczor et al. [163] applied the elaborated protocol for modeling GPCR dimers to construct a model of dopamine D_2_ receptor homodimer in inactive conformation, intended to study interactions of bivalent antagonists. The best scored model was an asymmetric model with TM4-TM5-TM7-TM1 interface.

It should be stressed that despite a number of reports about their pharmacological importance as drug targets, the design of drugs acting through GPCR dimers is still a challenge. The most common approach is the design of bivalent ligands. Bivalent ligand which contain two pharmacophoric molecules connected by a linker are, however, not drug-like due to high molecular mass. The design of bivalent ligands is based on the assumption that they will interact with two monomers in the dimer. It requires however, that the linker between molecules forming a bivalent ligand has appropriate length. Molecular docking of bivalent ligands to GPCR complexes is connected with a number of technical difficulties and it cannot be made automatically by docking software. Thus, it can be overcome by e.g., manual finding of an appropriate pose and refining it with docking software [163,164].

## 7. Summary and Perspectives

In recent years, there have been a number of examples of successful use of docking to GPCRs. Some of these reports concerned very subtle phenomena, like allosteric modulation or signaling bias, where a low affinity ligands in exposed binding pockets were in play, or when a very accurate reproduction of the docking mode was essential [24,30,31,124]. On the one hand, it is encouraging for further applications of in silico studies, but on the other, some of the most successful of the reported methods, like all-atom molecular dynamics or induced fit docking require considerable computational resources, which restricts both their availability and applicability. However, in most cases, core-saving simplifications of algorithms or protocols avoiding the most computationally-expensive steps can be applied with no loss on quality [31,134,138,139]. Moreover, code optimization and applications of GPU-based architectures further increase applicability of such methods [139,165]. With growing availability of both docking software and the necessary hardware, careful processing by the user becomes one of the most important, limiting steps in small-molecule docking. Therefore, understanding of the algorithm nature and proper preparation of both ligand and protein models becomes crucial, together with consideration of water molecules and the receptor activation states. Meanwhile, protein-protein and peptide-protein docking remains a very challenging task, since their flexibility makes it difficult to consider the relevant range of conformational space. It’s unfortunate, since in recent years awareness of the importance of GPCR dimers and oligomers have grown, and possibility of efficient modeling of such complexes could result in number of advances, e.g., in targeting binding sites at the edge of monomers, or targeting dimer interfaces. However, the considerable rate of developments in the field suggests, that increase in efficiency of these methods will be noticeable in the nearest future.

## Figures and Tables

**Table 1:** Brief summary of the capabilities of available software for predicting water displacement and energetic contributions in docking and screening.

Software	Description
GRID	Developed in 1985 by Goodford. It aims to identify favorable interaction sites for probe molecules and water is one of available probes. GRID energy is computed by summing Lennard-Jones, electrostatic and hydrogen bonding interactions.
HINT	Developed by Kellogg and co-workers. Combination of Hydrophobic Interactions (HINT) and the geometric descriptor rank into a statistically robust method to detect water molecules for consideration in protein-ligand docking and structure-based drug discovery techniques.
Superstar	SuperStar is capable of combining IsoStar propensity maps in order to calculate hotspots in protein binding sites.
JAWM	Developed by Michel and co-workers. Just Add Water Molecules (JAWM) procedure applies a double decoupling technique to compare the energetic cost of removing a water molecule from the bulk and from a binding site.
WaterMap	WaterMap (Schrödinger) is based on explicit solvent molecular dynamics simulations followed by statistical thermodynamic analyses of water clusters based on inhomogeneous solvation theory.
Water PMF	Developed by Zheng and co-workers. Water PMF applies the potential of mean force on 3946 nonredundant high resolution crystal structures.
Water Flap	Fingerprint for Ligands and Proteins (FLAP), based on the GRID Molecular Interaction Fields enables the user to carry out molecular docking automatically calculating the probability of crystallographic or predicted water molecules to be retained upon ligand binding to the protein target.
Gold	In GOLD water molecules are allowed to spin and toggle on and off. Toggling a water molecule on introduces an entropic penalty to the scoring function which needs to be offset by forming hydrogen bonds to the protein and the ligand. If the hydrogen bonds formed by the water molecules does not offset the entropic penalty introduced by turning the water molecule on then the water molecule will be deselected for (turned off) during the genetic algorithm run.
DOCK	Flexible-receptor docking method considering displaced water and retained water states with variable water position.
FlexX	Algorithmic approach, called the particle concept, for integrating the placement of single water molecules in the docking algorithm of FLEXX.
AutoDock	Hydration force field accounting for the entropic and enthalpic contributions of discrete waters to ligand binding.
Glide	Statistics about the number of hydrogen bonds formed by polar and apolar groups.
